# Genetic ablation of purine salvage in *Cryptosporidium parvum* reveals nucleotide uptake from the host cell

**DOI:** 10.1073/pnas.1908239116

**Published:** 2019-09-30

**Authors:** Mattie C. Pawlowic, Mastanbabu Somepalli, Adam Sateriale, Gillian T. Herbert, Alexis R. Gibson, Gregory D. Cuny, Lizbeth Hedstrom, Boris Striepen

**Affiliations:** ^a^Center for Tropical and Emerging Global Diseases, University of Georgia, Athens, GA 30602;; ^b^Department of Cellular Biology, University of Georgia, Athens, GA 30602;; ^c^Department of Pathobiology, School of Veterinary Medicine, University of Pennsylvania, Philadelphia, PA 19104;; ^d^Department of Pharmacological and Pharmaceutical Sciences, College of Pharmacy, University of Houston, Houston, TX 77204;; ^e^Department of Biology, Brandeis University, Waltham, MA 02454;; ^f^Department of Chemistry, Brandeis University, Waltham, MA 02454

**Keywords:** parasite, purine, *Cryptosporidium*, diarrhea, nucleotide

## Abstract

The apicomplexan parasite *Cryptosporidium* is a leading global cause of severe diarrheal disease and an important contributor to early-childhood mortality. Waterborne outbreaks occur frequently, even in countries with advanced water treatment capabilities, and there is currently no fully effective treatment. Nucleotide pathways are attractive targets for antimicrobial development, and several laboratories are designing inhibitors of these enzymes as potential treatment for *Cryptosporidium* infections. Here we take advantage of newly available molecular genetics for *Cryptosporidium parvum* to investigate nucleotide biosynthesis by directed gene ablation. Surprisingly, we found that the parasite tolerates the loss of classical targets including dihydrofolate reductase-thymidylate synthase (DHFR-TS) and inosine monophosphate dehydrogenase (IMPDH). We show that thymidine kinase provides a route to thymidine monophosphate in the absence of DHFR-TS. In contrast, only a single pathway has been identified for *C. parvum* purine nucleotide salvage. Nonetheless, multiple enzymes in the purine pathway, as well as the adenosine transporter, can be ablated. The resulting mutants are viable under normal conditions but are hypersensitive to inhibition of purine nucleotide synthesis in their host cell. *Cryptosporidium* might use as-yet undiscovered purine transporters and salvage enzymes; however, genetic and pharmacological experiments led us to conclude that *Cryptosporidium* imports purine nucleotides from the host cell. The potential for ATP uptake from the host has significant impact on our understanding of parasite energy metabolism given that *Cryptosporidium* lacks oxidative phosphorylation and glycolytic enzymes are not constitutively expressed throughout the parasite life cycle.

## Significance

Nucleotides are the activated building blocks for DNA and RNA as well as the major form of energy in all living organisms. Cells need to synthesize nucleotides to grow. Interference with this synthesis is thus widely used to treat infections and cancers. Here we discover through genetic experimentation that the parasite *Cryptosporidium* surprisingly does not require purine nucleotide synthesis. This may reflect the presence of a second novel purine salvage pathway. Alternatively, we propose that the parasite has evolved to import purine nucleotides, making purine salvage dispensable. Nucleotide import may also allow *Cryptosporidium* to steal energy from host cells. This finding has far-reaching consequences for the development of treatments for this important cause of diarrheal disease.

The protozoan parasite *Cryptosporidium* is one of the most important infectious causes of diarrheal disease. Large-scale epidemiological studies linked *Cryptosporidium hominis* and *parvum* infection with severe disease and death in small children (1), but also found significant adverse effects in milder cases (2). *Cryptosporidium* infection has a complex and profound interaction with early-childhood malnutrition, a key risk factor for and a potentially lasting consequence of this parasite infection (3, 4). Currently neither fully effective drug treatments nor prophylactic vaccines are available (3, 5). The parasite invades enterocytes in the apical brush border of the intestinal tract, where it develops a set of unique cellular structures, including a pedestal formed with host cell actin and a membranous feeder organelle thought to provide access to host metabolites.

*Cryptosporidium* is a member of the phylum Apicomplexa, which includes *Plasmodium* and *Toxoplasma*, the causes of malaria and opportunistic and congenital infection, respectively. Comparing the genome of *C. parvum* with that of *Plasmodium falciparum* or *Toxoplasma gondii* reveals dramatic gene loss (6). *C. parvum* has a diminutive genome (9 Mbp) encoding about 3,500 genes. Metabolism is one important aspect of parasite biology that experienced loss and streamlining in *C. parvum*. This parasite lost the apicoplast, a chloroplast-like organelle responsible for the synthesis of isoprenoids, fatty acids, and heme (7). While a mitochondrion is still present, it has lost its genome along with the tricarboxylic acid (TCA) cycle and oxidative phosphorylation. Curiously, glycolytic enzymes are not constitutively expressed throughout the parasite life cycle, suggesting a differentiated energy metabolism that is not fully understood (8). Beyond these organellar functions, *C. parvum* has also lost numerous anabolic pathways typically localized to the cytoplasm. *C. parvum*’s core metabolic map (9) is remarkably similar to the minimal sets found in obligate intracellular bacteria like *Chlamydia* and *Rickettsia*, parasites that make very little on their own but instead commandeer from the host.

This metabolic minimalism suggested that the remaining pathways and enzymes are essential to the growth of the organism and thus promising targets for drug development. The recent advent of CRISPR/Cas9–based genome engineering for *Cryptosporidium* (10) allowed us to put this hypothesis to a rigorous experimental test. Here we use gene targeting to disrupt multiple enzymes in the parasite’s purine and pyrimidine nucleotide pathways, two of which, dihydrofolate reductase-thymidylate synthase (DHFR-TS) (11) and inosine monophosphate dehydrogenase (IMPDH) (12), are currently pursued as targets for the treatment of cryptosporidiosis. Loss of these enzymes comes without apparent fitness cost, but renders the parasites keenly dependent on host cell nucleotide synthesis. Using inhibitors and tracers, we probe this metabolic host–parasite relationship and conclude that *C. parvum* imports purine nucleotides. This finding has far-reaching consequences in that it suggests that *C. parvum* could be an energy parasite.

## Results and Discussion

### *C. parvum* Tolerates Loss of Dihydrofolate Reductase-Thymidylate Synthase.

**Fig. 1.**
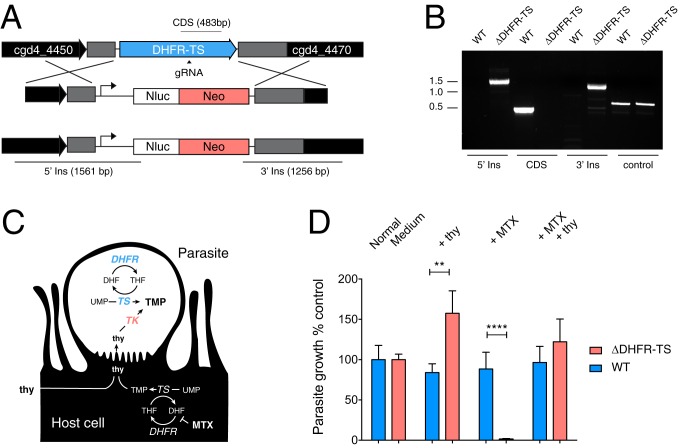
Genetic ablation of *C. parvum* dihydrofolate reductase-thymidylate synthase. (*A*) Schematic map of the locus of *C. parvum* DHFR-TS replaced by homologous recombination with the Nluc-Neo marker conferring resistance to paromomycin. CDS, coding sequence; gRNA, guide RNA. (*B*) PCR mapping of WT and ablated locus (ΔDHFR-TS) -producing amplicons illustrated in *A*. (*C*) Highly simplified metabolic map of host and parasite thymidine monophosphate (TMP) synthesis. Both can synthesize TMP from UMP, or salvage thymidine. (*D*) Parasite growth in HCT-8 cells over 48 h measured using an image-based assay using VVL staining (12) arbitrarily set to 100% for each strain to the growth observed in normal culture medium. Media were supplemented with 10 μM thymidine (+thy), 1 μM methotrexate (+MTX), or both. *n* = 6; error bars show SD. Note that WT growth (blue) remains unchanged while growth of ΔDHFR-TS (red) is significantly impacted by manipulation of host cell thymidine levels, suggesting that the mutant relies on import of thymidine from the host. Significance was determined using a 1-way ANOVA with Tukey’s multiple comparisons test (**P < 0.01, ****P < 0.0001).

In plants and many protists, dihydrofolate reductase (DHFR) and thymidylate synthase (TS) are encoded by a single gene resulting in the expression of a fused dual-activity enzyme (DHFR-TS). Thymidylate synthase is the dominant user of reduced tetrahydrofolate and therefore this arrangement may allow for substrate channeling and/or coordinated expression of 2 important cell cycle-regulated enzymes (13). In *P. falciparum* and *T. gondii*, DHFR-TS activity is required for growth and the DHFR domain is a clinically validated drug target. In contrast, *C. parvum* is resistant to antifolate treatment and we have suggested that the horizontal gene transfer of a thymidine kinase (TK) might have rendered DHFR-TS dispensable (10, 14). To directly test this hypothesis, we targeted the DHFR-TS locus for ablation in *C. parvum*. Sporozoites were electroporated with a plasmid expressing Cas9 and a U6 promoter-driven guide RNA designed to produce a double-strand break in the middle of the DHFR-TS–coding region. A DNA cassette encoding a NanoLuciferase-neomycin resistance (Nluc-Neo) marker flanked by 1,000 bp matching the up- and downstream sequences of the DHFR-TS gene was targeted to the locus for replacement of the endogenous gene by homologous double cross-over (Fig. 1*A*). Interferon (IFN) γ knockout mice were infected with transfected sporozoites, and paromomycin was provided as a selection agent in the drinking water of the mice. Luciferase-positive oocysts were apparent 9 days postinfection (*SI Appendix*, Fig. S1) and were passaged continuously through multiple sets of mice under continued selection, producing infection and oocyst shedding. We mapped the DHFR-TS locus of wild-type (WT) and ΔDHFR-TS–modified parasites using PCR of genomic DNA extracts and multiple primer sets. Amplicons are consistent with a double homologous cross-over at the 5′ and 3′ ends of the gene and most importantly the loss of the entire DHFR-TS–coding sequence (Fig. 1*B*). We conclude that DHFR and TS activities are dispensable in *C. parvum*.

### *C. parvum* Lacking DHFR-TS Depends on Host-Supplied Thymidine.

We infected intestinal epithelial cell monolayers (human ileocecal adenocarcinoma line HCT-8) with ΔDHFR-TS or the parental WT parasites and quantified intracellular growth after 48 h of culture. Both strains proliferated normally, but we noted that growth of ΔDHFR-TS was enhanced when the medium was fortified with 10 μM thymidine (Fig. 1*D*). This suggests that the parasite can forgo its own synthesis if thymidine is provided by the host (see Fig. 1*C* for a schematic overview of thymidine metabolism in host and parasite). To test this further, we examined the growth of both strains in the presence of methotrexate (MTX), a potent inhibitor of mammalian dihydrofolate reductase that blocks host thymidine monophosphate (TMP) synthesis by starving the reaction for reduced folate (15). Growth of ΔDHFR-TS parasites was ablated under these conditions but could be fully rescued by providing excess thymidine in the medium. Methotrexate treatment was conducted at a commonly used dosage range (15) and treatment had no effect on growth of WT parasites in the presence or absence of thymidine. We conclude that DHFR-TS and TK provide *C. parvum* with redundant routes to TMP and loss of either enzyme renders the parasite hypersensitive to inhibition of the other. Our experiments do not formally distinguish rescue of the mutant by uptake of host-derived thymidine or TMP. However, our previous experiments demonstrated that ΔTK parasite strains can no longer be labeled with 5-ethynyl-2′-deoxyuridine (EdU), a thymidine analog (10). Thus, the parasite cannot take up thymidine/EdU nucleotides from the host cell.

### *C. parvum* Inosine Monophosphate Dehydrogenase Is Dispensable.

**Fig. 2.**
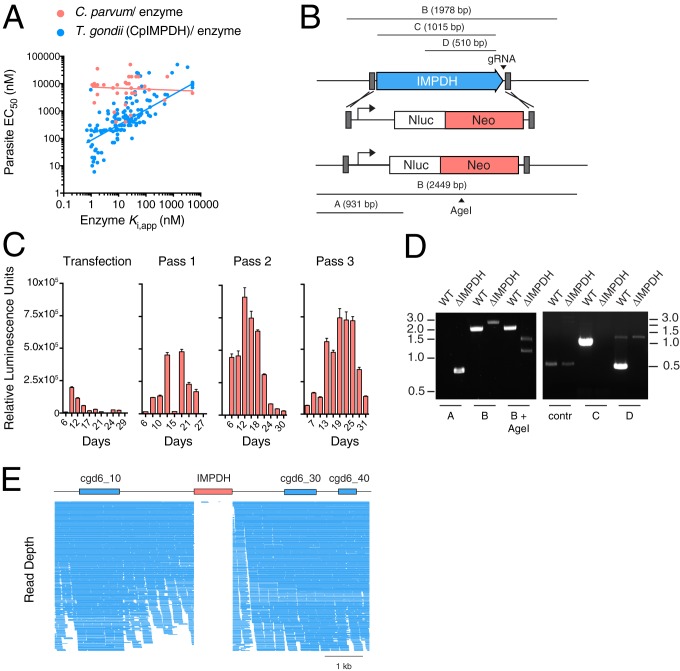
Targeting of the *C. parvum* inosine monophosphate dehydrogenase locus. (*A*) Comparison of the impact on parasite growth and enzyme inhibition of multiple series of IMPDH inhibitors (each symbol represents one compound; see *Results* for reference). Note that for *T. gondii* engineered to depend on *C. parvum* IMPDH [HXGPRT deletion and IMPDH replacement (12)], potency of compounds against the organism correlates with that of the recombinant enzyme over 4 orders of magnitude (*r*^2^ = 0.17, *P* < 0.0001); no correlation is observed for *C. parvum*. (*B*) Modified targeting procedure using 50-bp flanking sequences (gray). PCR products used to map the WT and insertion locus are labeled “A” to “D.” To further validate ablation, amplicon B produces a pattern distinguishing WT and ΔIMPDH when treated with the restriction enzyme *Age*I. (*C*) NanoLuciferase activity was measured in the feces of mice infected with transfected parasites. The emerging paromomycin-resistant parasites were passed multiple times in animals, demonstrating the viability of the strain. Samples were pooled from 4 to 6 mice in triplicate; error bars are SD. (*D*) PCR mapping of WT and ablated locus (ΔIMPDH) -producing amplicons illustrated in *B*. (*E*) WT and ΔIMPDH *C. parvum* were subjected to whole-genome sequencing. Individual sequence reads mapping to IMPDH and surrounding loci in ΔIMPDH are shown. Note gene loss.

**Fig. 3.**
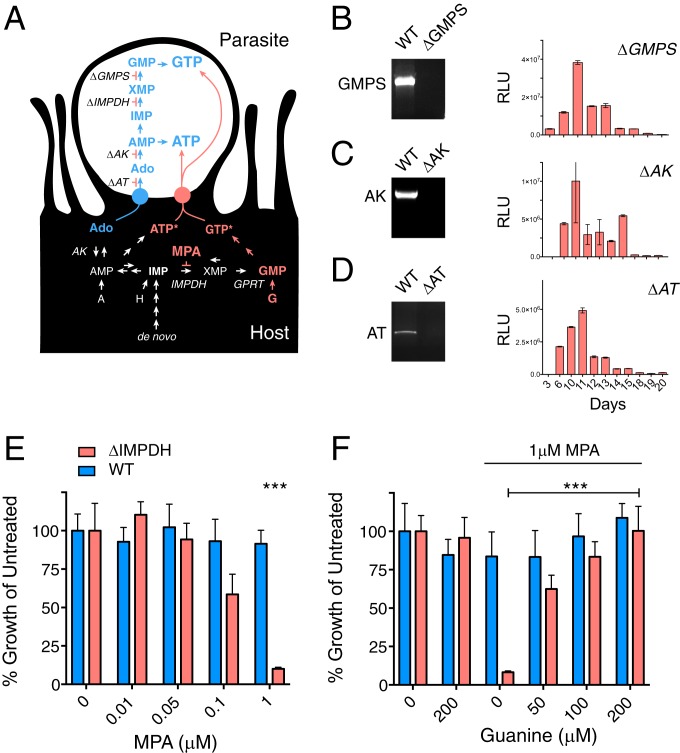
*C. parvum* tolerates loss of purine synthesis, which renders it dependent on host-derived purine nucleotides. (*A*) Simplified schematic representation of human host and *C. parvum* parasite purine metabolism. The parasite purine salvage pathway is shown in blue; the enzymes and transporter ablated in this study are indicated. The proposed parasite nucleotide uptake pathway is highlighted in red. The host can synthesize purines de novo or salvage different bases and nucleosides. Mycophenolic acid (MPA) blocks all routes to GMP except guanine phosphoribosyltransferase (GPRT). A, adenine; Ado, adenosine; G, guanine; H, hypoxanthine. *Note that it is yet unknown whether the presumptive nucleotide transporter imports tri- (as shown here), di-, monophosphates, or all of them. (*B*–*D*) GMPS, AK, and AT can be ablated. PCR of WT and respective mutant strains demonstrating loss of coding sequence. Please refer to *SI Appendix*, Figs. S2–S4 for full details on the validation of these 3 deletion mutants including mapping controls and whole-genome sequencing. NanoLuciferase measurements (reported as relative luminescence units; RLUs) from mouse feces showing that all strains are viable and readily infect mice. Samples were pooled from 4 to 6 mice in triplicate; error bars are SD; representative graph from 1 of 3 biological repeats. (*E*) *C. parvum* WT and ΔIMPDH parasites were cocultured with human host cells and subjected to MPA treatment over a range of concentrations (growth was measured by luciferase assay). Note the hypersensitivity of ΔIMPDH at 1 µM (***P < 0.001). (*F*) Growth of ΔIMPDH treated with MPA is rescued by addition of guanine to the medium (***P < 0.001). (*E* and *F*) Unpaired, 2-tailed *t* test with Welch’s correction. *n* = 6; 1 of 3 biological repeats is shown; error bars are SD.

While pyrimidine synthesis is redundant in *C. parvum*, genome analysis predicts a nonredundant and highly streamlined pathway for purine nucleotide synthesis based on uptake and phosphorylation of adenosine to AMP (6, 14), followed by 3 further steps leading ultimately to the production of guanosine monophosphate. Inosine monophosphate dehydrogenase is the penultimate step in this pathway (see Fig. 3*A*). *C. parvum* IMPDH appears to be a particularly attractive potential drug target due to its evolutionary origin through horizontal gene transfer from bacteria, because it is the rate-limiting step in this pathway, and because it is clinically validated in a variety of diseases (14, 16–18). *Cp*IMPDH-selective inhibitors were identified via high-throughput screening (18), and subsequent medicinal chemistry optimization increased the potency of enzyme inhibition by factors of 10^3^ to 10^4^. However, this improvement resulted in only modest improvement of antiparasitic activity (18–22). In contrast, a *T. gondii* strain engineered to depend on *C. parvum* IMPDH (12) showed pronounced susceptibility to these compounds in culture that correlated with potency of enzyme inhibition (Fig. 2*A*). This disparity could reflect differences in compound uptake or host cell metabolism, or unforeseen differences in the purine salvage pathways of the 2 parasites.

To evaluate the importance of IMPDH directly in *C. parvum*, we attempted to ablate the IMPDH gene. We used a modified protocol employing a repair cassette generated by PCR featuring only 50 bp of homologous upstream and downstream sequence to target the locus (Fig. 2*B*). These sequences were introduced using synthetic oligonucleotides, avoiding the need for plasmid cloning. Luciferase-expressing parasites readily emerged after mice were infected with transfected sporozoites. Transgenic parasites showed infection and growth in multiple subsequent passages (Fig. 2*C*) and mutant parasites showed no obvious growth defect in mice or HCT-8 cells. PCR mapping demonstrated insertion of the marker cassette into the locus and loss of the IMPDH-coding sequence (Fig. 2*D*).

We considered that the locus might carry multiple copies of the gene or might have undergone duplication or rearrangement in a way that would preserve IMPDH at a different location in the genome. We performed whole-genome sequencing of the ΔIMPDH strain and determined it to be >99% identical to the published genome for the *C. parvum* IOWA strain. Genomic alignment of the ΔIMPDH strain demonstrated ablation of the locus consistent with the PCR results and found no evidence for additional sequences encoding IMPDH in the genome of the mutant strain (Fig. 2*E*). We conclude that IMPDH is not essential for *C. parvum* growth in tissue culture or mice.

### *C. parvum* Purine Salvage Can Be Ablated at Multiple Steps of the Pathways.

The finding that ΔIMPDH mutants are viable was surprising, as loss of IMPDH should deprive the parasite of XMP and subsequently the undoubtedly essential GTP (6, 9, 14). We considered that the parasite may harbor a yet-to-be-described enzyme that could synthesize XMP using an alternative mechanism and thus sidestep the loss of IMPDH. To explore this hypothesis, we focused on guanosine monophosphate synthase (GMPS), the last enzyme in the pathway that converts XMP to GMP (Fig. 3*A*). We targeted GMPS for genetic ablation, this time introducing an additional yellow fluorescent protein (YFP) reporter in the gene replacement cassette. Transgenic parasites were obtained, they were fluorescent, showing insertion of the marker (*SI Appendix*, Fig. S2 *A* and *B*), and PCR mapping demonstrated loss of the GMPS-coding sequence (Fig. 3*B* and *SI Appendix*, Fig. S2*C*). ΔGMPS parasites grew without apparent defect in vitro and in vivo, mirroring the findings for ΔIMPDH.

We next explored the importance of AMP synthesis in *C. parvum*. In *P. falciparum*, AMP is synthesized from IMP by the successive and essential activities of adenylosuccinate synthase and adenylosuccinate lyase (23–25). *T. gondii* can synthesize AMP following the same route from IMP or alternatively by phosphorylation of adenosine through adenosine kinase (AK) (26, 27). The required adenosine is imported by a specific adenosine transporter (AT) (28). The *C. parvum* genome does not encode adenylosuccinate synthase or lyase, but homologs of the *T. gondii* AK and AT are present (14, 26). Biochemical studies using recombinant enzyme have demonstrated that *C. parvum* AK phosphorylates adenosine to AMP (28). We targeted AK and AT for ablation in *C. parvum* and found that both genes can be removed without apparent detriment to the parasite (Fig. 3 *C* and *D*; also see *SI Appendix*, Figs. S3 and S4 for full mapping and genome sequencing). Thus, we conclude that adenosine salvage is not essential. In total, we ablated the transporter and 3 of the 4 enzymes of the annotated purine nucleotide pathway of *C. parvum* (the fourth enzyme was not attempted). We conclude that this pathway is dispensable for parasite growth.

### Loss of IMPDH Sensitizes *C. parvum* to Inhibition of Host Purine Synthesis.

Like all living cells, the parasite needs a source of ATP and GTP to thrive. How is this satisfied in the absence of the salvage pathway? While it is possible that this parasite harbors novel enzymes and transporters that permit purine salvage independent of the canonical pathway, we hypothesized that *C. parvum* may be one of the rare intracellular pathogens capable of stealing purine nucleotides from the cytoplasm of their host cell. Transporters capable of this remarkable feat were first described for *Rickettsia* (29). This model predicts that the purine synthesis knockouts are reliant on nucleotide import from the host cell. To test this, we investigated parasite growth in host cells treated with mycophenolic acid (MPA), a potent inhibitor of human IMPDH. In humans, IMPDH is crucial to obtain GMP through de novo purine synthesis as well as for salvage of adenosine, adenine, or hypoxanthine (30) (see the schematic in Fig. 3*A*). *Cryptosporidium* IMPDH and human IMPDH are phylogenetically divergent (14, 17) and differences in their structure and kinetic mechanism render the parasite enzyme 1,000-fold more resistant to MPA (*K*_i_ = 9.3 μM) when compared with the 2 human enzymes [*K*_i_ = 11 and 6 nM (31–35)]. We treated infected cultures with MPA over a range of concentrations and measured parasite growth. We note that MPA at these concentrations is routinely used to select transgenic strains for the related parasite *T. gondii* (36). As predicted, WT parasites were resistant to MPA treatment, but ΔIMPDH parasites were hypersensitized in a dose-dependent fashion, leading to a full growth arrest at 1 μM (Fig. 3*E*; *P* value < 0.0001). Human cells can circumvent a block in IMPDH through the activity of guanine phosphoribosyltransferase (GPRT), an enzyme not present in *C. parvum* (6, 14). We repeated the MPA treatment and supplemented the medium with exogenous guanine, the substrate of GPRT, up to 200 μM. Guanine complementation rescues growth of the MPA-treated ΔIMPDH mutant in a dose-dependent fashion (Fig. 3*F*; *P* value < 0.0001). We conclude that *C. parvum* can import guanine nucleotides from host cells. In wild-type parasites, guanine nucleotide uptake is redundant with the parasite’s own purine nucleotide pathway. However, mutants lacking purine synthesis rely entirely on purine nucleotide import from the host.

### Adenosine Analogs Trace Host Nucleotide Import into *C. parvum*.

**Fig. 4.**
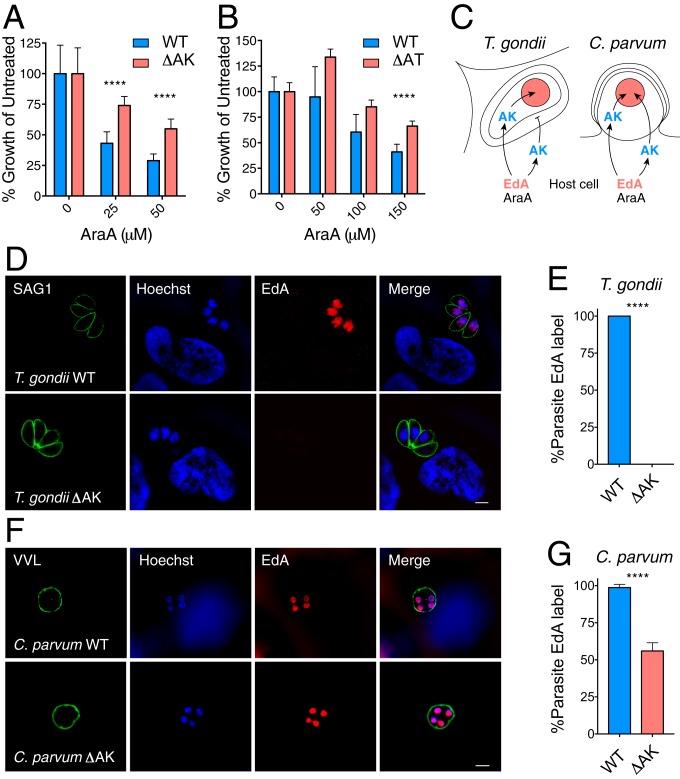
*C. parvum* imports adenosine nucleotides. (*A* and *B*) WT and ΔAK (*A*) or ΔAT (*B*) parasites were grown in HCT-8 cells in the presence of different concentrations of adenosine arabinoside (AraA) and growth was measured by NanoLuciferase assay (WT: NanoLuciferase expression site integrated adjacent to TK gene causing no gene disruption). Unpaired, 2-tailed *t* test with Welch’s correction, ****P < 0.0001; 1 of 3 biological repeats is shown; error bars are SD; (*A*) *n* = 12, (*B*) *n* = 6. (*C*) Schematic outline of nuclear EdA labeling in *T. gondii* and *C. parvum*. (*D* and *F*) Cultures were infected with *T. gondii* (*D*) or *C. parvum* (*F*) for 12 h and then incubated with 10 µM EdA for 12 h, followed by fixation and permeabilization. (Scale bars, 5 μm.) EdA was labeled by click chemistry (red), DNA was stained with Hoechst (blue), and parasites were detected using an antibody to SAG1 or using *V. villosa* lectin (VVL; both green). Images shown were acquired by structured illumination superresolution microscopy. (*E* and *G*) Quantification of EdA labeling (*n* = 100; 3 biological replicates; error bars show SD; ****P < 0.0001). *T. gondii* but not *C. parvum* nuclear labeling with EdA is abolished in ΔAK.

The *T. gondii* adenosine kinase and adenosine transporter were initially discovered in genetic experiments selecting for resistance to the prodrug adenine arabinoside (AraA) (26–28). *T. gondii* AK loss-of-function mutants showed 50-fold resistance compared with WT (37) and AT mutants no longer incorporated radiolabeled adenosine (28). We have previously demonstrated that AraA is a substrate for *C. parvum* AK and that growth of *C. parvum* is susceptible to AraA in culture (14, 38). In contrast to *T. gondii*, we found that loss of AK or AT produced only moderate resistance to AraA (Fig. 4 *A* and *B*) of a 1.4- and 2.1-fold increase in the EC_50_, respectively (across 3 independent biological experiments).

Sustained susceptibility suggests that AraA is phosphorylated by the human host AK and then AraA nucleotide is imported by the presumptive *C. parvum* purine nucleotide transporter. Alternatively, AraA could poison the host cell and thus indirectly affect parasite growth.

To distinguish between these 2 alternatives, we sought to build a positive assay for purine nucleotide import using 7-deaza-7-ethynyl-2′-deoxyadenosine (EdA), an adenosine analog. EdA is activated by AK and incorporated into DNA, where it can be labeled fluorescently using azide-alkyne click chemistry (39). In *T. gondii* cultures pulsed for 12 h with 10 µM EdA, all parasite nuclei incorporated EdA (red fluorescence, Fig. 4 *D* and *E*). This labeling depends entirely on parasite AK, as the nuclei of *T. gondii* ΔAK mutants do not label with EdA. We performed comparable experiments with *C. parvum* and again noted robust EdA labeling of parasite nuclei in WT parasites (Fig. 4 *F* and *G*). In contrast to *T. gondii*, we found only moderate reduction of EdA labeling the *C. parvum* ΔAK strain. We conclude that in *C. parvum*, but not *T. gondii*, there are 2 pathways leading to adenosine (and EdA) incorporation: one where adenosine is activated by AK in the parasite cytoplasm, and a second path where adenosine is activated by host AK in the host cell cytoplasm followed by import of an EdA nucleotide into the parasite.

## Conclusion

We used CRISPR/Cas9–driven genetic engineering of *C. parvum* to ablate multiple genes involved in nucleotide metabolism, pathways that were widely presumed to be critical for parasite survival. Remarkably, these strains were viable. While nucleotide synthesis has proven an excellent target for antimicrobial drugs including for several Apicomplexa, the fact that these enzymes, in particular DHFR-TS and IMPDH, can be ablated in *Cryptosporidium* suggests that their chemical inhibition will likely not result in effective treatments. These findings inspire renewed caution when predicting the essentiality of presumptive drug targets from metabolic maps solely derived by bioinformatic analysis.

It appears that the unique horizontal gene transfer of TK provides *C. parvum* with an alternative route to TMP and thus renders DHFR-TS dispensable. Alternatively, TMP import from the host could satisfy this need and current evidence neither proves nor refutes the existence of such a transporter. However, parasites ablated for DHFR-TS (this study) or TK (10) are similarly hypersensitive to the inhibition of the alternative enzyme. This argues that TMP must be synthesized in the parasite and that therefore *C. parvum* likely lacks a mechanism to import TMP. In contrast, *C. parvum* does not have known alternative routes for purine synthesis. Nonetheless, the purine salvage enzymes are dispensable as demonstrated by multiple knockout strains reported here. This is in stark contrast to *T. gondii* and *P. falciparum*, where purine salvage is essential (25, 40) and a firm requirement for tracer labeling (Fig. 4 *D* and *E*).

We cannot formally exclude the possibility that *C. parvum* may harbor a novel, noncanonical purine salvage pathway that relies on 2 new enzymes and a new transporter. However, such a pathway does not readily explain our host-directed inhibitor and fluorescent tracer experiments as well as uptake of purine nucleotides from the host cell. Why would *C. parvum* import purine but not pyrimidine nucleotides when both are required for DNA and RNA synthesis? We propose energy parasitism as the potential evolutionary root of this difference. Under this hypothesis, *C. parvum* imports purine nucleotides because it relies on the host cell ATP pool to satisfy its energy needs. Such transporters were first described in *Rickettsia* (29) and *Chlamydia* (41). Microsporidia, highly reduced intracellular fungal pathogens, were similarly shown to import purine nucleotides, and the genes for these transporters appear to have been acquired by horizontal gene transfer of the ancestral rickettsial genes (42) or evolved from other members of the major facilitator superfamily (43). Acquisition of ATP transport in these organisms is associated with a significantly reduced energy metabolism. This is most pronounced in the microsporidian *Enterocytozoon bieneusi*, which lost its central carbon metabolism including glycolysis (44). *C. parvum* energy metabolism has similarly sustained extensive loss, most prominently the TCA cycle and oxidative phosphorylation (9). Transcriptional profiling of *C. parvum* life-cycle stages revealed differential expression of the glycolytic pathway with pronounced induction in female gametes and during sporogony (8). Glycolysis may not be essential for intracellular growth, but rather be required for extracellular parasite stages, including oocyst persistence and the motility of invasive sporozoite and merozoite stages. Further work is required to directly test the importance of glycolysis and energy metabolism in different phases of the parasite’s development.

What is the mechanism of *C. parvum* purine nucleotide import? Earlier biochemical studies on *P. falciparum* suggested a link between host and parasite ATP levels (45); however, no specific transporter has been identified. Genome searches using a variety of algorithms have so far failed to identify direct homologs of the nucleotide transporters identified in Microsporidia in the genomes of *Cryptosporidium* species. *Cryptosporidium* may have adapted a novel type of nucleotide transporter or, alternatively, coopted organellar transporters from its mitochondrion or chloroplast. Further work is needed to discover the identity of this purine nucleotide transporter.

## Materials and Methods

### Materials.

Mycophenolic acid, vidarabine monohydrate (adenine arabinoside), methotrexate, and thymidine were purchased from Sigma-Aldrich, and guanine was purchased from Acros Organics.

### *C. parvum* Mutant Construction.

The DHFR-TS gene (cgd4_4460) was targeted using a guide RNA consensus sequence located 373 bp upstream of the stop codon. Regions of 980 bp upstream and 1,024 bp downstream of the coding sequence were used to target replacement with the dual reporter–drug selection cassette containing a NanoLuciferase-neomycin resistance fusion protein driven by *C. parvum* regulatory elements (10). DNA constructs for deletion of other enzymes were designed in a similar manner. The IMPDH gene (cgd6_20) was targeted using a guide RNA 32 bp downstream of the stop codon; 50 bp directly upstream and downstream of the coding sequence was used for recombination. GMPS (cgd5_4520) was targeted using a guide RNA 294 bp downstream of the start codon and regions of homology were derived from the 50 bp directly upstream of the guide sequence and 50 bp before the stop codon. This replaced 1,707 bp of the gene with the Nluc-Neo selection cassette and a YFP reporter. Similarly, AK (cgd8_2370) was targeted using a guide RNA 56 bp downstream of the start codon with flanks from the first 50 bp of the coding region and a region 80 bp downstream of the stop codon, and AT (cgd2_310) was targeted for deletion using a guide RNA 15 bp upstream of the stop codon and 50 bp directly upstream and downstream of the ORF, replacing the entire coding sequence. All primers are listed in *SI Appendix*, Table S1.

### *C. parvum* Mutant Isolation.

*C. parvum* were purchased from Bunchgrass Farms and sporozoites were electroporated with a plasmid containing Cas9 and the appropriate guide RNA along with a PCR product carrying the replacement cassette as detailed above. Transfected sporozoites were then used to infect mice. Female IFN-gamma knockout mice (B6.129S7-*Ifng*^*tm1Ts*^/J; JAX 002287) aged 6 to 8 wk were treated with an antibiotic mixture in the drinking water for a week before infection, normal drinking water the day of infection, and subsequently with paromomycin (10). For sporozoite infection via gavage, each mouse was gavaged twice with 100 μL saturated sodium bicarbonate in ultrapure water followed immediately by gavage with transfected sporozoites in cold PBS. Fecal samples (pooled from the cage) were collected and assayed as previously described (NanoGlo Luciferase Assay Kit and GloMax luminometer; Promega) (10). Fecal DNA was isolated using the Zymo Fecal DNA MicroPrep Kit (Zymogen) and PCR was performed to map insertions. Transgenic oocysts were purified from fecal material as previously described (10). All animal experiments were reviewed and approved by the Institutional Animal Care and Use Committee of the University of Georgia (A2016 01-028-Y1-A4) or the University of Pennsylvania (806292).

### Whole-Genome Sequencing.

Genomic DNA was isolated from 10^6^ excysted oocysts of WT, ΔIMPDH, ΔAK, or ΔAT using the Qiagen DNeasy Blood and Tissue Kit. Libraries were prepared with Nextera XT Kits (Illumina) and 150-bp paired end reads were collected on a MiSeq system (Illumina). Reads were aligned to the reference genome for *C. parvum* IOWA (CryptoDB-43 assembly, EuPathDB) using the Burrows–Wheeler Aligner (46) and sequence variants were called using the Genome Analysis Toolkit haplotype caller with a ploidy of 1 (47). Raw sequence files are available through the NCBI (BioProject ID: PRJNA543286).

### Cell Culture.

Human ileocecal colorectal adenocarcinoma cells (HCT-8; ATCC CCL-244) were cultured at 37 °C, 5% CO_2_ (10). *T. gondii* was grown in human foreskin fibroblasts as described (14).

### Luciferase Parasite Growth Assays.

Bleach-treated oocysts (20,000 per well) were cocultured with 70% confluent HCT-8 in 96-well cultures. For luciferase assays, media were removed at 48 h and replaced with 100 μL lysis buffer (NanoGlo Luciferase Assay System; Promega); plates were incubated at 37 °C, 5% CO_2_ for 15 min; 100 μL substrate mixture was added to each well and mixed; and finally, samples were transferred to white plates and read in a luminometer. For image-based assays, samples were stained using *Vicia villosa* lectin (VVL; Vector Labs) and DAPI; 4 × 4 tiled images were collected at 40× using an automated microscope and parasites and host cell nuclei were quantified using a macro written for ImageJ (48).

### Nucleotide Labeling.

At 12 h postinoculation, EdA was added (10 µM final concentration), incubated for 12 h, and fixed. EdA incorporation was detected using the Click-iT Plus EdU 594 Imaging Kit (Thermo Fisher). Coverslips were stained with Hoechst (1 mg/mL) and VVL was conjugated to fluorescein for *C. parvum* or a monoclonal antibody to SAG1 for *T. gondii* (1:1,000; gift of Jean-Francois Dubrementz, University of Montpellier, Montpellier, France; ΔAK *T. gondii* were a gift of David Roos, University of Pennsylvania). Images were acquired on a DeltaVision OMX SR or on a Leica DM6000 at the Penn Vet Imaging Core and processed using ImageJ.

## Supplementary Material

Supplementary File
