# Supplementary material for: Genetic ablation of purine salvage in Cryptosporidium parvum reveals nucleotide uptake from the host cell
Source: Proc Natl Acad Sci U S A. 2019 Sep 30;116(42):21160–5. doi: 10.1073/pnas.1908239116 (PMC6800313; doi:10.1073/pnas.1908239116)
Supplement: Supplementary File [file pnas.1908239116.sapp.pdf]

PNAS
www.pnas.org

Supplementary Information for:

Genetic ablation of purine salvage in *Cryptosporidium parvum* reveals nucleotide uptake from the host cell

*Mattie C. Pawlowic, Mastanbabu Somepalli, Adam Sateriale, Gillian T. Herbert, Alexis R. Gibson, Gregory D. Cuny, Lizbeth Hedstrom, and Boris Striepen**

*Corresponding author
Phone: +1-215-5739167
Email: striepen@upenn.edu

This PDF file includes:

Tables S1
Figures S1 to S4

www.pnas.org/cgi/doi/10.1073/pnas.1908239116

Table S1. Primers used in this study

Purpose/Region	Primer Name	Sequence (5' to 3')
**DHFR-TS KO cloning**	DHFR 5'flank F	aggccgctctagaactagtggatccTTCTGGAATTTATTACACCAGC
	DHFR 5'flank R	gtttccccaccgcggTTTAAAAGTTCTGAAATTCACAAC
	DHFR 3'flank F	attcttctgactgcagTCTATTATTTATAAAATATCCATAGTAGTCAG
	DHFR 3'flank R	tctagagaataggaacttcaccggtCTACTACTCTCATCACTGC
	Cas9 DHFR guide F	gttgGCCATTTGAGATAGAGCAGA
	Cas9 DHFR guide R	aaacTCTGCTCTATCTCAAATGGC
**DHFR-TS diagnostic PCR**	DHFR 3' R	GTCCCGATCTACACGAAATTATACCAACG
	DHFR 5' R	CCTTCCGATTACAGCAGGTGCTATG
	Nluc R	CTGTCGCCAGTCCCCAACG
	Neo F	GGTATCGCCGCTCCCGATTC
	IntDHFR R	GAATTTGAGTTGCGGAAATGGACGTG
	IntDHFR F	GGTTTAGGGCATAGAGAGGAGAATGAT TTG
	TKgene F	ATGGCAAAATTATACTTTTACTATTCAGCAATGAATGC
	TKgene R	TTAGAAATTGTATTCTTCACAATTAATTATATGATGTTTTCTGC
**IMPDH KO cloning**	IMPDH g#1 F	gttggATTTAATATCACGGAAGGGG
	IMPDH g#1 R	aaacCCCCTTCCGTGATATTAAATc
	IMPDH g#1 F Gibson	ttaatataaaaactgaaaggttaagtgttggATTTAATATCACGGAAGGGGgttttagagctaGAAAtagcaagttaaaat
	IMPDH g#1 R Gibson	attttaacttgctaTTTCtagctctaaaacCCCCTTCCGTGATATTAAATccaacacttaacctttcagtttttatattaa
	IMPDH KO Homology F	gcgccaaattcctgttatatgataaatattaattagatatcgactatttcTGGGGAAACTAAATATACTGAAATTCGGTAGATTCT
	IMPDH KO Homology R	gctcatatgatttaaatttcaaaatgaaaaaaccaaaataaatttacaatttaatatcacggaagggg AATTAAGATAAAAAGAAAAACTTAATCGATACTATCC
	IMPDH HA F	ggattaagagaaagtcatgttcatgatgttgaaatcgtaaaagaagtaatgaattatagtaaaCCTAGGTACCCGTACGACGTCCCGGAC
**IMPDH diagnostic PCR**	IMPDH Screen F	GTTCAATCCTTGCTGGCACTGAAG
	IMPDH Screen R	CAAGTATTTGTAATTCTAAGTTCATC
	IMPDH F #1	GAGTGGTAGTGGGGATAGATATTTCCAAGAGAAAAGACCTG
	IMPDH F #2	GATGTAATTGTTGGGAATGTTGTAACAGAAGAAGCAAC
	IMPDH F #3	GGTTCCAAATTATTCAGAGGTACTACCAAGAGAAGTTAGC
	IMPDH R #1	CCCTCCTAGTCTCGCCATTCCTACTGCCATTAAATGTTCTGTTACTGTATCC
	IMPDH R #2	GAGGAACACCAACTCCTGCAACAATTCTTGTGGTACAAATACTTCCTGGACC
	IMPDH R #3	GCAGGATCTTAGTCCTCCAACAAGCTGATATACTACACCTTCCATTTCACC
	IMPDH R #4	CAAACTGGTGACTATATTTGTTTAACAAAC
	IMPDH Scr F #1	GCTTAGTGGATTGGCGCCAACATATTATTTTATTCAAGTG
	IMPDH Scr F #2	GTTTAGACAGAATCAACGTGAAGTGATTCTATATTTAATG
	IMPDH Scr R #1	CATCTCTACCATACGTAAGATATGGCTTTC
**GMPS KO Cloning**	GMPS g#1 F	gttgGCATAACCAAATTCACCAAT
	GMPS g#1 R	aaacATTGGTGAATTTGGTTATGC
	GMPS AYA KO Homology F	GGACATTATATTGTTAATATTGGTTATGGTGGTCAAGTACATAAAGCACCCAGTGGGCTTCAGAATGAGTTGGTTATAAACAGTAATA
	GMPS AYA KO Homology R	TCCCAGCCAAATGTTGCTGGTGGTTTATTTGTTACATCAAAATATATTGCAATTAAGATAAAAAGAAAAACTTAATCGATACTATCCTACACGCCACG
**GMPS diagnostic PCR**	GMP Up Scr 1	GCTGGAATAGAAGGACGATCTACATC
	GMP Up Scr 2	GAACTTGCGGAGACCAGCTTC
	GMP ORF F	CAAGTATGGATGAGTCATCAAGATG
	GMP ORF R	GTCATAACATCTTCAGAACATACAGGACG
	GMP Down Scr 1	GAATCAGAAGGCAATACATTATCAAAGG
	GMP Down Scr 2	GGATATCATGATGGAACAGGTTGTGG
**AK KO Cloning**	8_2370 g#1F	gttgGGCATCTCCTGATAGAATAA
	8_2370 g#1R	aaacTTATTCTATCAGGAGATGCC
	AK KO F	ATGAGGGGAAAGAAAATATTTGGAATGTGCAACCCGATCTTGGATATAGTGtggggaaactaaatatactgaaattcggtagattct
	AK KO R	GGAAATGAATAGTAAAAATTACATGCAGGTATAAAAATTGTATTACGTGGTGaattaagataaaaagaaaaacttaatcgatactatcc
	AK YFP KO F	ATGAGGGGAAAGAAAATATTTGGAATGTGCAACCCGATCTTGGATATAGTGcagtgggcttcagaatgagttggttataaacagtaata
**AK diagnostic PCR**	AK Up Scr 1	GGGTGTGGGATGATAGTTAGATGCC
	AK Up Scr 2	CGCAAAAGGCGGCAACTCTCTGC
	AK ORF F	AGGGCTTAAGATAGGGTCAACGAC
	AK ORF R	CACAGCCAACATTTTGAGCAACATTAGAGGC
	AK Down Scr 1	GCCTTGCAAGCTCATGAACACAC

	AK Down Scr 2	GCTGGAATAGCCGGCATCCTCTC
**<u>AT KO Cloning</u>**	2_1310 g#1F	gttgGTGGAAGTAGAAGAAGTTTG
	2_1310 g#1R	aaacCAAACTTCTTCTACTTCCAC
	AT KO F Homology	ggaaataatatatttgaaagattttatcacaatgaaatctgaaagactagTGGGGAAACTAA ATATACTGAAATTCGGTAGATTCT
**AT diagnostic PCR**	AT KO R Homology	gatcatattacgattccatagattgttattccaatattagggttgtaaataatcAATTAAGATAAA AAGAAAAACTTAATCGATACTATCC
	AT ORF F2	ggaatgggatttattcaatatgttc
	AT ORF R2	gtctggatgtggttagaatccagagaagagaagg
	AT Up F2	gagaacctagcttttacaaactttgaagg

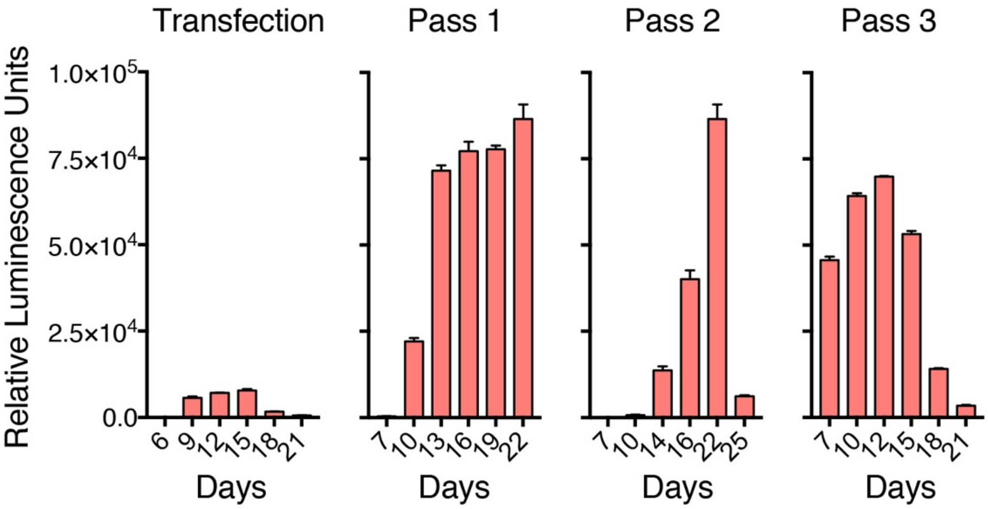

Supplementary Figure 1. *C. parvum* dihydrofolate reductase-thymidylate synthase (DHFR-TS) deletion mutants are viable. ∆DHFR-TS parasites emerged from drug selection and were continuously passed to naive mice under paromomycin treatment. Nanoluciferase measurements of collected mouse feces is shown. One passage represents fecal sample pooled from a cage of infected mice. Bars illustrate the average and error bars are standard deviation.

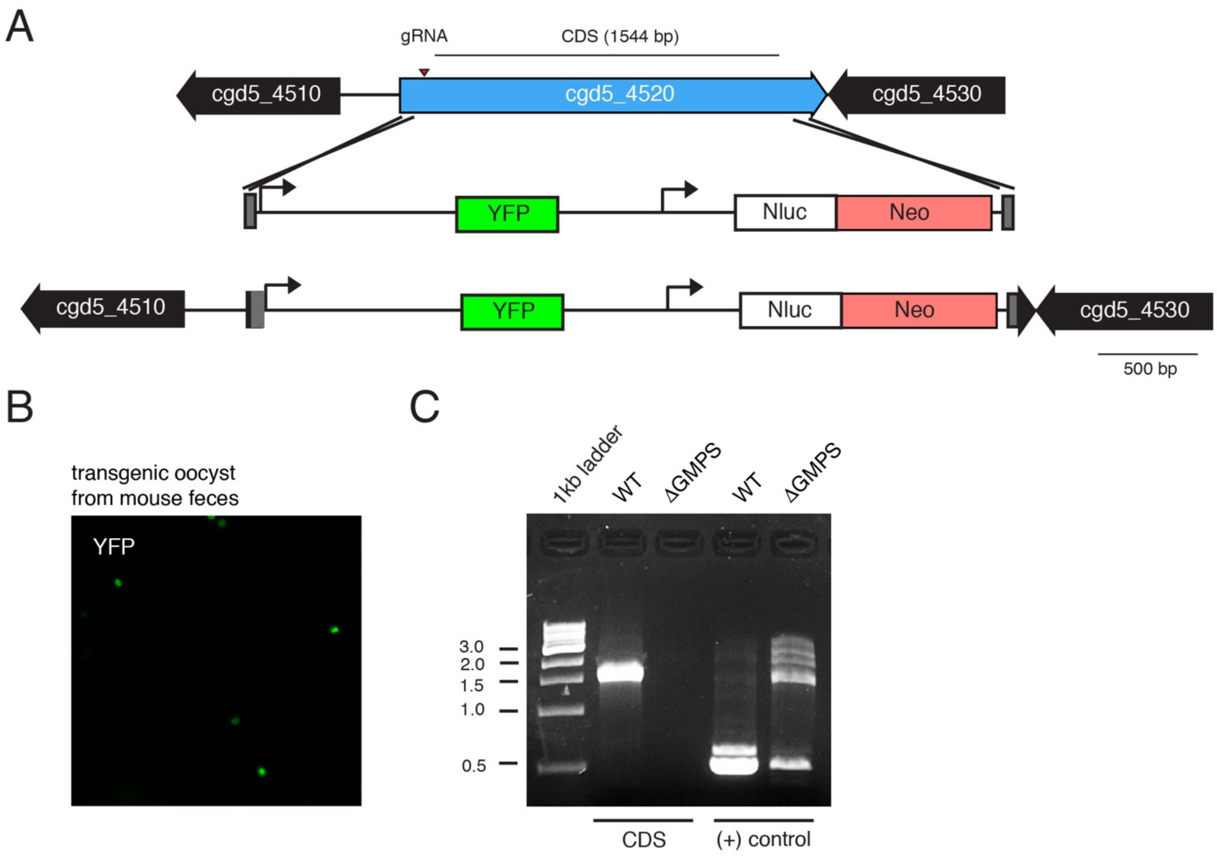

Supplementary Figure 2. Genetic ablation of *C. parvum* guanine monophosphate synthase (GMPS). (A) Schematic map of the locus of *C. parvum* GMPS replaced by homologous recombination with a yellow fluorescent protein (YFP) reporter and Nluc-Neo marker conferring resistance to paromomycin. (B) Epifluorescence microscopy demonstrates YFP expression of purified ΔGMPS oocysts. (C) PCR mapping of wild type (WT) and ablated locus (ΔGMPS) producing amplicons illustrated in (A).

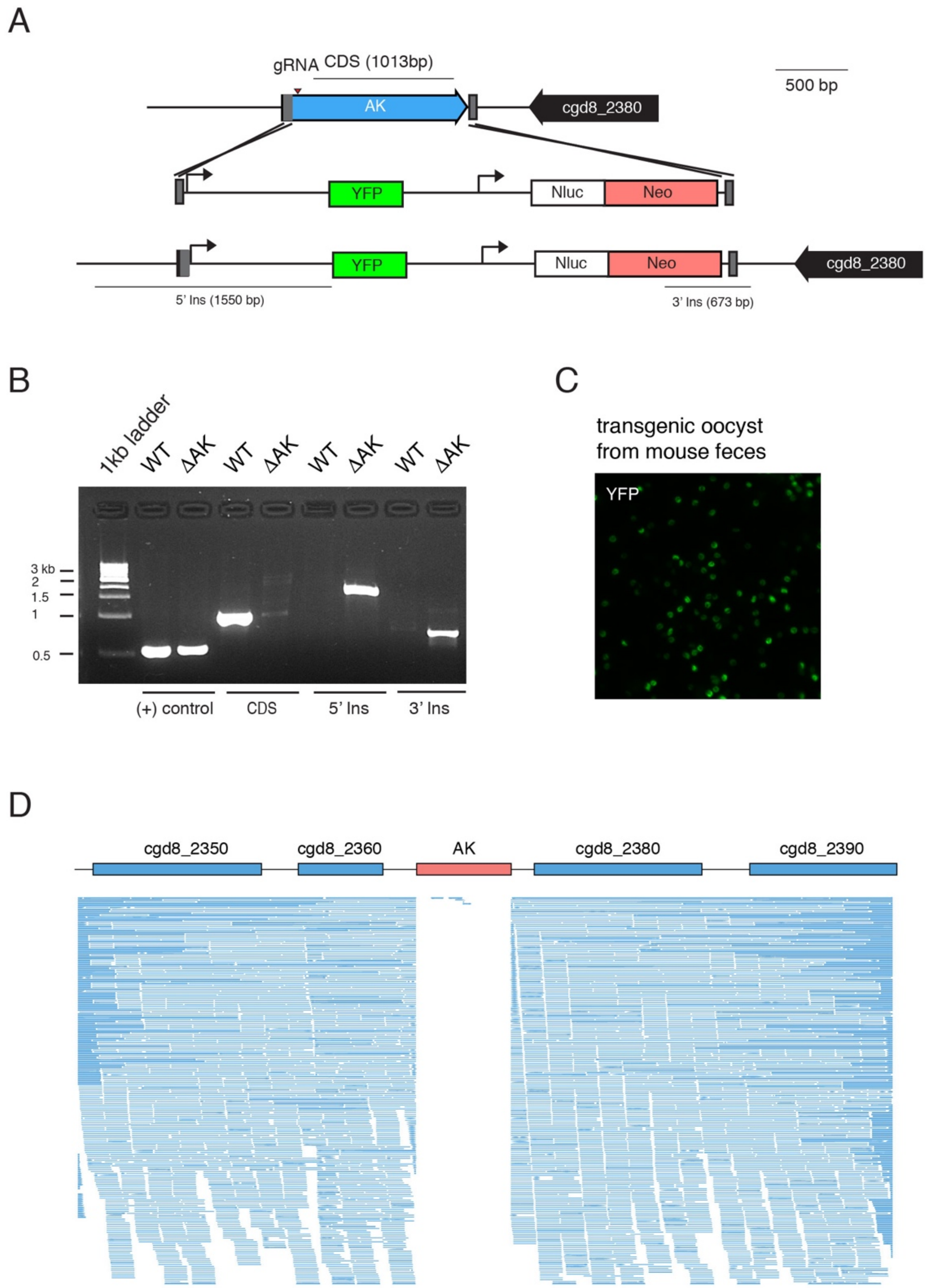

Supplementary Figure 3. Genetic ablation of adenosine kinase (AK). (A) Schematic map of the locus of *C. parvum* AK replaced by homologous recombination with the YFP reporter and Nluc-Neo marker conferring resistance to paromomycin. (B) PCR mapping of wild type (WT) and ablated locus (ΔAK) producing amplicons highlighted in (A). (C) Epifluorescence microscopy demonstrates YFP expression of purified ΔGMPS oocysts. (D)The genome of ΔAK was sequenced and reads aligning to the AK locus and its surrounding genes are shown here. Note loss of sequencing reads that align to the AK coding sequence.

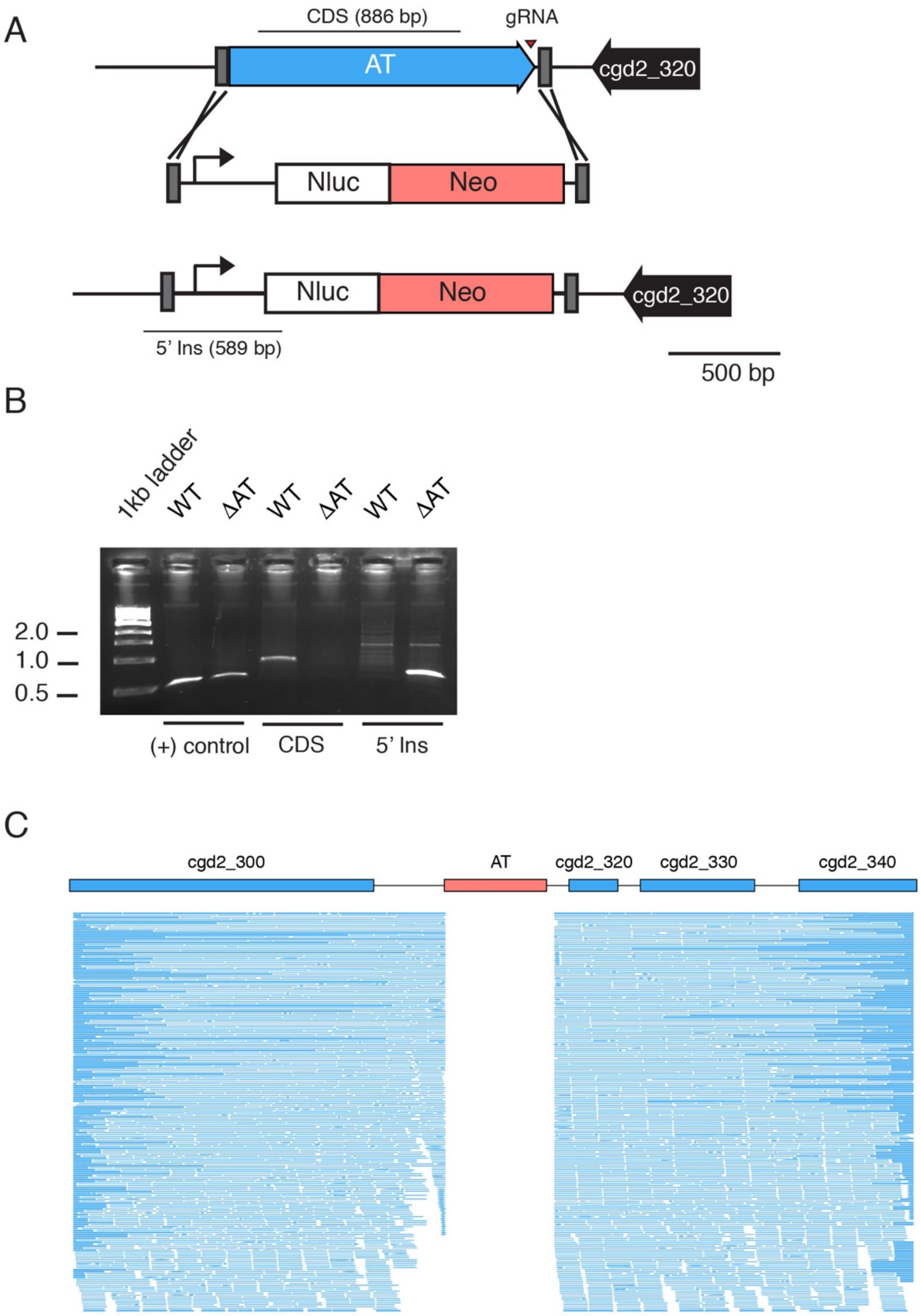

Supplementary Figure 4. Genetic ablation of the adenosine transporter (AT). (A) Schematic map of the locus of *C. parvum* AT replaced by homologous recombination with the Nluc-Neo marker conferring resistance to paromomycin. (B) PCR mapping of wild type (WT) and ablated locus (ΔAT) produces amplicons illustrated in (A). (C) The genome of ΔAT was sequenced and reads aligning to the AT locus and its surrounding genes are shown here. Note loss of sequencing reads that align to the AT coding region.
